# Rental assistance impacts on diabetes: Insights from a longitudinal mixed-methods analysis

**DOI:** 10.1016/j.ssmqr.2026.100817

**Published:** 2026-06-29

**Authors:** Whitney Denary, Denise Esserman, Penelope Schlesinger, Kasia J. Lipska, Andrew Fenelon, Danya E. Keene

**Affiliations:** a Yale School of Public Health, Department of Social and Behavioral Sciences, New Haven, CT, USA; b Yale School of Public Health, Department of Biostatistics, New Haven, CT, USA; c Yale School of Medicine, Department of Internal Medicine, New Haven, CT, USA; d Yale University, Section of Endocrinology, New Haven, CT, USA; e University of Minnesota-Twin Cities, School of Public Health, Minneapolis, MN, USA

## Abstract

The U.S. faces a persistent shortage of affordable housing, forcing many low-income individuals with chronic illnesses such as diabetes to navigate trade-offs between housing, healthcare, and basic needs essential for disease management. Rental assistance may alleviate these pressures by increasing housing stability, reducing financial strain, and enabling safer medication storage and food preparation. However, only a fraction of eligible households receive assistance, and long waitlists create a natural comparison group for examining health impacts. This mixed-methods, two-and-a-half-year longitudinal study utilizes data from the Project ReSIDe Study, which followed 113 adults with diabetes who were on a waitlist for rental assistance in Connecticut at baseline. Using a linear mixed-effects model, we examined within-person changes in glycemic control (HbA1c) as participants transitioned into rental assistance. We did not observe a significant difference in changes in HbA1c between those who obtained rental assistance vs. those who did not across follow-ups. Qualitative interviews with a subsample of 30 participants revealed substantial heterogeneity in experiences of transitioning from waitlists to rental assistance, which may help explain these null findings. For some participants, rental assistance supported diabetes management by preserving or improving access to medical care and increasing autonomy in day-to-day diabetes management. For others, diabetes management was hindered by disrupted medical care after relocation or by inadequate kitchen conditions, including lack of refrigeration, stoves, or pest-free environments. These findings suggest that efforts to improve health through rental assistance may require complementary attention to housing quality, residential stability, and continuity of medical care.

## 1. Introduction

The United States is experiencing a persistent and severe shortage of affordable rental housing, forcing low-income households to make difficult trade-offs between rent, basic necessities, and healthcare. In no state does a full-time minimum-wage job provide sufficient income to rent a market-rate two-bedroom apartment without spending more than 30% of household income on rent, and the majority of poor renters spend over 50% of their income on housing (Colón-Bermúdez et al., 2025). These high housing cost burdens contribute to eviction, residential instability, homelessness, and substandard living conditions, challenges disproportionately affecting Black, Hispanic, and other racial/ethnic minority households because of longstanding racialized housing policies and discrimination in housing markets (Hanson & Hawley, 2011; Swope et al., 2022; Whittaker et al., 2025). At the same time, affordable housing challenges have important implications for population health, through both physical housing conditions and psychosocial pathways, including housing-related stress, insecurity, and lack of control over one's living environment (Padgett, 2007; Pollack et al., 2010; Shaw, 2004; Smith et al., 2024; Swope & Hernández, 2019).

These issues may be especially consequential for adults with chronic health conditions, including diabetes—a prevalent and costly disease that most heavily burdens low-income individuals and historically marginalized communities, who are also at greatest risk of housing instability (Gaskin et al., 2014; Hill-Briggs et al., 2021). Diabetes requires complex daily management, including medication adherence, glucose monitoring, dietary regulation, physical activity, and regular medical care, all of which can be disrupted by unstable or unaffordable housing (Berkowitz et al., 2025; Mosley-Johnson, Walker, Thakkar, et al., 2022; Vijayaraghavan et al., 2011). Inadequate glycemic control increases the risk of short-term emergencies and long-term complications, making stable housing conditions potentially critical for disease management and future health outcomes (Berkowitz et al., 2018, 2025; Sharan et al., 2023).

Federal rental assistance programs, administered primarily through the U.S. Department of Housing and Urban Development (HUD), aim to reduce housing cost burden by capping rent at 30% of household income for families earning below 50% of area median income, thereby improving housing affordability, stability, and autonomy. Given that market-rate housing is largely inaccessible to low-income households and fewer than one in four eligible families receive assistance—with average wait times of 28 months—rental assistance represents a scarce but potentially powerful structural intervention that could mitigate housing-related stressors and improve health (Acosta & Gartland, 2021; Colón-Bermúdez et al., 2025).

A growing body of research suggests rental assistance may shape diabetes-related outcomes. Qualitative studies indicate assisted housing can alleviate health-demoting financial stress related to diabetes expenses and improve individuals' diabetes-related routines and social support by providing the autonomy and residential stability that comes with having a place of one's own, rather than living in doubled-up or overcrowded housing (Keene, Henry, et al., 2018). Quantitative research using linked national HUD and federal health datasets has further demonstrated associations between rental assistance and diabetes outcomes. One study investigating low-income renters diagnosed with diabetes found that those with rental assistance had lower odds of cost-related medication nonadherence compared to those not receiving rental assistance (Sanjeevi et al., 2025). Similarly, quasi-experimental studies comparing individuals receiving rental assistance to those who would receive it in two years (a “pseudo-waitlist”) have found that rent-assisted individuals have lower odds of undiagnosed diabetes, and residents specifically in project-based housing were less likely to have uncontrolled diabetes (HbA1c ≥ 9.0), a pattern not observed among voucher recipients (Fenelon et al., 2022; Gonzalez-Lopez et al., 2024). These findings demonstrate potential health benefits for tenants with diabetes who receive federal rental assistance.

Despite these advances, important gaps remain in understanding how rental assistance affects diabetes management. Existing quantitative studies have primarily relied on national administrative datasets and pseudo-waitlist designs, which are powerful for estimating average population-level associations but provide limited information about the timing of transitions into rental assistance, within-person changes in glycemic control, or the lived experiences underlying observed outcomes. In addition, much of the prior work has focused on diabetes diagnosis or the prevalence of uncontrolled diabetes, rather than examining changes in HbA1c over time among individuals as they move into assisted housing. As a result, less is known about the mechanisms through which rental assistance may facilitate—or, in some cases, hinder—day-to-day diabetes management.

To address these gaps, this study draws on mixed-methods, longitudinal data from the Project ReSIDe Study, which followed adults with diabetes who were initially on a rental assistance waitlist to observe their transitions into assisted housing over a two-and-a-half-year follow-up. We examine whether obtaining rental assistance is associated with changes in glycated hemoglobin (HbA1c) over time and use in-depth qualitative interviews to illuminate the pathways through which housing conditions and medical access may facilitate or hinder diabetes management. By combining quantitative and qualitative evidence, we move beyond asking whether rental assistance improves diabetes outcomes on average to ask how, for whom, and under what conditions it shapes glycemic control.

## 2. Methods

### 2.1. Sampling and recruitment

The mixed-methods analyses presented here utilize longitudinal survey, biomedical, and interview data from the Project ReSIDe Study. This study examined the relationship between rental assistance and health outcomes for individuals with diabetes. Eligible participants were required to (1) be at least 18 years old; (2) have a self-reported diagnosis of type 1 or type 2 diabetes; (3) be on a waitlist for income-based rental subsidies; and (4) reside in Connecticut at the time of enrollment. Staff determined eligibility during a one-on-one phone screening, during which potential participants provided information on their health, demographics, and housing waitlist status, including the type of waitlist and the year of application.

Participant enrollment occurred between November 2020 and February 2022. The research team utilized multiple strategies to identify and recruit potential participants. Recruitment methods included posting flyers in community settings, using digital marketing on Facebook, advertising on the exterior of local buses, circulating flyers to participants of previous studies, conducting in-person recruitment at a diabetes clinic, listing the study on our institution's open research registry, presenting to community ambassadors, and engaging a community advisory board to circulate information about the study.

This study was approved by the Yale University IRB, and all participants provided written informed consent prior to enrollment.

### 2.2. Data collection and measures

We collected three primary forms of data during this longitudinal study: survey, objective health, and interview. Survey and health data from the entire cohort of 113 participants were collected every six months over a two-and-a-half-year period, resulting in six waves of data collection. Participants received $75 compensation for each wave of survey and health data completion.

The interview data were collected from a purposive subsample of 30 participants selected to participate after the first survey follow up. To ensure a sufficient representation of experiences transitioning into rental assistance, half of the sample (n = 15) had already reported receiving rental assistance at the time of selection. Over the course of the study, an additional five interview participants leased up with rental assistance, resulting in 20 interview participants leased up with rental assistance, and 10 who remained on the waitlist. Qualitative participants completed four rounds of semi-structured interviews conducted six months apart, and received $50 in compensation for each interview.

Throughout the study, participants contributed 618 survey observations, with high retention: 93% completed the first follow-up, 87% the second, 85% the third, 87% the fourth, and 84% the final follow-up. Attrition over the study period reflected the structural and health vulnerabilities of the study population, with some participants unable to be located, others temporarily unavailable due to illness, hospitalization, or incarceration, and eight participants dying during the study period.

#### Survey data:

The surveys were programmed and administered using Qualtrics, covering topics related to their housing and health, including diabetes symptoms and management, rental assistance, economic well-being, housing, neighborhood, general and mental health, food security and nutrition, substance use, and social support. Participants were given the option to take the survey independently (on their own device or in the study office) or to have a staff member read the survey questions to them (either during an in-person appointment or over the phone). Participants were also given the option to take the survey in English or Spanish. Below, we describe the survey variables we include in the analyses for this paper.

##### Rental Assistance Variable:

The survey asked participants if they were currently receiving rental assistance and, if so, which specific type. This question included a comprehensive list of all types of local and available rental assistance and used multiple terms to help ensure participants could identify these types. For example, Housing Choice Vouchers were also listed as Section 8 vouchers, given that applicants often use the latter term. Participants who reported receiving rental assistance were asked to indicate the month and year they began receiving it.

For the purpose of the analyses presented in this paper, participants were classified as receiving rental assistance at a given follow-up visit if they indicated they 1) had obtained rental assistance at least three months prior to their HbA1c collection, and 2) were listed on the lease for the rent-assisted unit. These criteria ensured sufficient time for rental assistance to potentially influence HbA1c outcomes, which reflect average glucose levels over the preceding two or three months.

Rental assistance types included: Public Housing, Project-Based Housing, Housing Choice Vouchers, Housing Vouchers for Veterans, Rental Assistance Program (RAP), Housing Access Service Organizations, Housing via Mental Health Service Providers, Ryan White, Housing Opportunities for Persons with AIDS, or Permanent Supportive Housing. These forms of rental assistance are non-time-limited and typically cap tenant rent at 30% of household income, meeting the federal definition of affordable housing, which differ from short-term or emergency forms of rental aid, such as the Emergency Rental Assistance (ERA) program that provided one-time payments during the COVID-19 pandemic to cover arrears or temporary income loss (Aiken et al., 2022). Because ERA does not adjust rent based on income or provide ongoing affordability, participants who had received ERA but remained on a waitlist for long-term subsidies were classified as “unassisted” and remained eligible for the study. At baseline, 10 participants reported receiving temporary ERA funding.

##### Individual Characteristics:

We collected demographic characteristics, including race (African American, White, and Other), ethnicity (Hispanic and non-Hispanic), and gender (man and woman). At baseline, participants also reported whether they had been employed in the past six months, had children under age 18 living at home, the year of their diabetes diagnosis, and had ever been incarcerated.

#### Objective health data:

Participants also supplied four health measures at each visit: HbA1c, weight, height, and blood pressure. All health data were collected within 30 days of the surveys. This analysis utilizes the primary health outcome, HbA1c, a clinical indicator of diabetes control that reflects average blood glucose over approximately two to three months. Participants could either (1) have their HbA1c measured by trained study staff using a portable finger-prick device, the A1CNow, or (2) provide documentation of HbA1c results from a healthcare provider obtained within 30 days of their study appointment (Arrendale et al., 2008). For participants choosing the first option, samples were collected either in the study office or at participants' homes. Early in the pandemic, health data measurements were collected outdoors to minimize COVID-19 exposure risk. Of note, the A1CNow device and multiple providers’ devices displayed a max output of 13. For participants who provided labs from a healthcare provider, HbA1c levels greater than 13 were rounded down for data consistency.

#### Interview data:

Participants in the qualitative subsample answered questions about their rental assistance trajectories, housing experiences, and their diabetes. Interviews were conducted either in person or via Zoom, recorded and transcribed verbatim, and lasted an average of 40 minutes in length (range 20–70 min). Three research staff members interviewed the same participants across all four interview waves (baseline and three follow-ups) to maintain consistency in rapport and narratives. Three participants completed their interviews in Spanish with a research staff member who was a native Spanish speaker. Interviewers utilized a semi-structured interview guide with open-ended questions and probes to balance predetermined topics of interest with themes introduced by participants related to their housing and health. All names used in this paper are participant-selected pseudonyms, and individual participant HbA1c values are rounded to the nearest half value to protect participants’ anonymity.

### 2.3. Quantitative statistical analysis

First, we compared the baseline characteristics of those who ultimately received rental assistance and those who did not (“Change in Rental Assistance”) using *t*-test (continuous) and chi-square tests (binary). These characteristics included participants’ age, race, ethnicity, and sex, as well as employment status, presence of children living at home, incarceration history, years since diabetes diagnosis, and baseline HbA1c.

Second, we used a linear mixed-effects model (LME) to examine the change in HbA1c across study waves relative to baseline, as a function of rental assistance status at each six-month follow-up. We hypothesized that participants who transitioned to rental assistance would experience greater reductions in HbA1c from baseline relative to those who remained on the waitlist. This analysis examined the association between rental assistance status and change in HbA1c, adjusting for baseline HbA1c and baseline demographic and clinical characteristics, including age, years since diabetes diagnosis, and incarceration history. We selected covariates based on prior literature linking these characteristics to both receipt of rental assistance and diabetes-related outcomes, serving as potential confounders of the relationship between housing assistance and HbA1c. We prioritized inclusion of variables that were plausibly confounders (i.e., associated with both exposure and outcome but not on the causal pathway), while excluding factors such as employment status and housing instability that may be influenced by receipt of rental assistance or function as mediators. This approach was intended to avoid overadjustment and post-treatment bias, which could attenuate the estimated effects of rental assistance.

This model included random intercepts for participants to account for within-person correlation across repeated measures and was estimated using restricted maximum likelihood (REML). This model takes the form: $Y_{ij}=\beta_{0}+\beta_{B}B_{i}+\beta_{H}H_{ij}+a_{i}+\varepsilon_{ij}$, where: $Y_{ij}$ is the HbA1c value for individual i at observation j, $B_{i}$ is a vector of baseline covariates (baseline HbA1c, age, years since diabetes diagnosis, and incarceration history), $H_{ij}$ indicates rental assistance status for individual i at observation j, $a_{i}$ is a random intercept for individual i, accounting for within-person correlation across repeated measures, and $\varepsilon_{ij}$ is the residual error term. These analyses were conducted using R (Version 4.4.2).

### 2.4. Qualitative analyses

Following a grounded theory approach, our analysis was an ongoing, iterative process that co-occurred with data collection (Corbin & Strauss, 2014). Interviewers wrote thematic summaries after each interview and analytic memos about developing concepts, and prior to each follow-up interview, the interviewer reviewed the participant's prior transcript to understand their trajectory over time. Throughout data collection and analysis, study team members met regularly to review interviews and discuss emerging themes. Once baseline interviews were completed, three team members open-coded a subset of transcripts and collaboratively developed an initial codebook through group discussion. A larger coding team then refined this codebook over a six-month period by applying it to small batches of transcripts in NVivo, discussing inconsistencies, redundancies, and code clarity, and revising the codebook through consensus meetings in multiple iterative rounds. After finalizing the codebook, research assistants independently applied it to the first set of transcripts in NVivo and resolved any discrepancies through discussion. The remaining transcripts, including follow-up interviews, were coded by a single coder, though the team continued to raise and resolve coding questions in regular meetings.

#### 2.4.1. Mixed-methods analysis

We sought to more fully understand how transitions to rental assistance could shape diabetes control by integrating our qualitative and quantitative data. To do so, we developed an analytic matrix for all 30 participants in our qualitative sample that organized participants’ housing trajectories alongside their HbA1c biomedical data and key qualitative themes from their interviews, enabling systematic comparison across cases while retaining the depth of individual narratives.

In constructing this matrix, the first author extracted and systematically reviewed coded excerpts related to housing conditions, housing transitions, rental assistance experiences, and diabetes management from the NVivo database. These excerpts were then situated within participants' broader narrative by reading full transcripts from all intervals, alongside wave-specific interview summaries to attend to continuity, change, and turning points in each participant's housing and health trajectory over time. Rather than treating excerpts as discrete pieces of data, this approach facilitated analysis of how participants made meaning of their housing experiences over time in relation to their diabetes management, daily routines, stress, and access to resources. In parallel, the first author compiled each participant's HbA1c values across all study waves and created individual longitudinal profiles of glycemic control. We examined the HbA1c trajectories in relation to key housing events—such as receiving a voucher, leasing up, relocating, experiencing housing instability, or resolving housing quality concerns—to assess how changes or stability in glycemic control corresponded with these transitions.

This structured integration of longitudinal narrative data and objective clinical indicators allowed us to identify convergences, divergences, and temporal sequencing between housing processes and diabetes outcomes. This iterative back-and-forth between narrative and objective health data supported a holistic, mixed-methods understanding of how housing-related processes intersected with clinical indicators of diabetes control.

## 3. Results

### 3.1. Quantitative findings

Of the 316 potential participants who responded about participating in the study, 167 were not eligible: 47 did not have diabetes, 52 already received rental assistance, and 68 were not on a waitlist. Of the 149 participants screened and eligible for the study, 113 enrolled and completed a baseline survey (Supplemental Fig. 1). Notably, our analytic sample was smaller than originally planned, limiting statistical power. Based on initial projections (75% retention and 70% uptake of rental assistance), a sample of 300 would have yielded approximately 157 transitions to rental assistance and 90% power to detect a 0.5% change in HbA1c (SD = 1%). The achieved sample size reduced the ability to detect effects of this magnitude and to perform subgroup analyses.

Baseline characteristics are presented in Table 1. The mean HbA1c was 8.0% (SD = 2.2; range: 4.2–13.0), with 28% having uncontrolled diabetes (HbA1c > 9%). During the study period, 42 participants (37.2%) began receiving rental assistance. Participants who eventually received rental assistance did not differ significantly from those who remained unassisted across baseline characteristics, including age, race, ethnicity, gender, employment status, history of incarceration, presence of children at home, or years since diabetes diagnosis. Baseline HbA1c levels were also similar between groups (mean 7.7% vs. 8.2%, p = 0.23). Nearly half of the sample (49%) reported a history of prior incarceration. We specifically asked about incarceration because existing literature demonstrates that involvement with the carceral system is associated with increased diabetes prevalence and disrupted healthcare access, as well as differential access to and eligibility for rental assistance and other housing supports (Keene, Rosenberg, et al., 2018; Purtle et al., 2020; Wildeman & Wang, 2017).

**Fig. 1.**
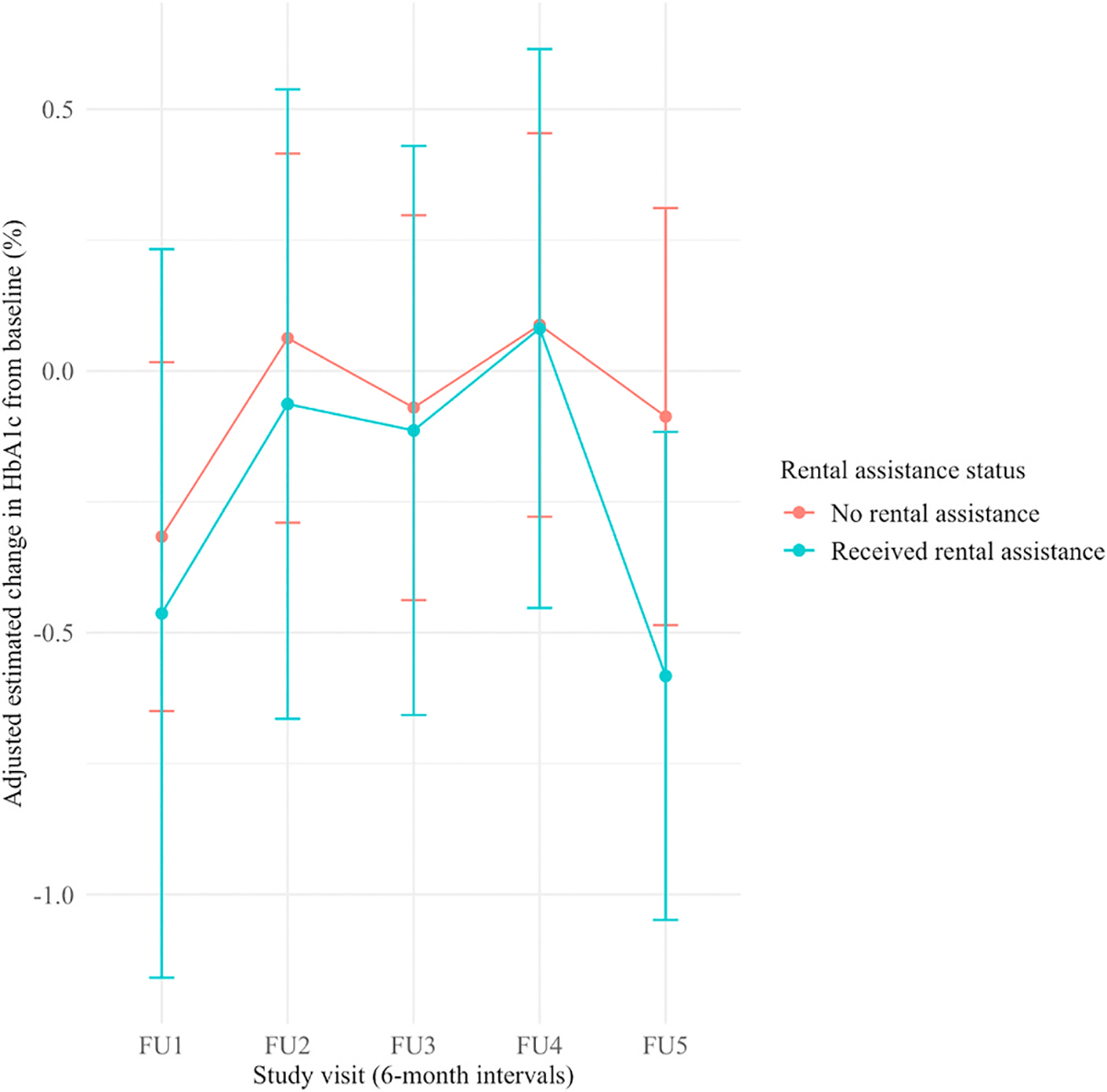
Adjusted Estimated Mean Change in HbA1c from Baseline by Rental Assistance Status **Note:** Study visits are at 6 month intervals. Estimates are adjusted marginal means from a linear mixed-effects model with random intercepts for participants, adjusted for baseline HbA1c, age, years since diabetes diagnosis, and incarceration history. Error bars represent 95% CIs. Rental assistance status reflects receipt for the past 3 months.

The linear mixed-effects model examined changes in HbA1c over time as a function of rental assistance status (Table 2). Across the first four follow-up waves, changes in HbA1c from baseline did not differ meaningfully between participants receiving rental assistance and those on the waitlist; between-group differences in HbA1c change were small and not statistically significant (Fig. 1). At the fifth follow-up, participants receiving rental assistance had a larger estimated reduction in HbA1c, although the between-group contrast at follow-up five did not reach statistical significance (β = 0.50; 95% CI: −0.06, 1.05; p = 0.079).

Higher baseline HbA1c values were significantly associated with greater reductions in HbA1c over time (β = −0.31; 95% CI, −0.42 to −0.21; p < 0.001). Older participants were more likely to experience a reduction of HbA1c (β = −0.02; 95% CI, −0.04 to 0.001; p = 0.06), but this association did not reach statistical significance. Duration of diabetes and incarceration history were not independently associated with HbA1c change.

### 3.2. Qualitative results

Our quantitative findings demonstrate a non-significant trend toward improvements in HbA1c following transitions into rental assistance. Our qualitative data allow us to examine the role of rental assistance acquisition in diabetes management, considering how it may facilitate or hinder glucose control. The sections below describe how the rental assistance shaped diabetes-related experiences and behaviors for our participants, including access to medical care, autonomy in diabetes management, and housing and kitchen quality.

#### 3.2.1. Medical care access

The act of acquiring rental assistance can simultaneously facilitate and hinder access to medical care, depending on where and how recipients move. Some participants reported that receiving rental assistance meant they had to move far away from their previously unsubsidized housing, where they had established medical care. Others found that using a voucher in their original housing meant fewer disruptions to their routine and continued access to medical care facilities.

Kim received a voucher in a town about 60 minutes away from her original housing. Following the move, Kim discontinued care with her previous medical team and intended to establish care with a new physician closer to her rent-assisted apartment. However, this took longer than intended, and Kim went over a year without a medical appointment. When asked about her diabetes, she said, “I think my diabetes is not going well because I have not found a doctor out here. I haven't been to the doctor. I haven't taken any medicine.”

This disruption in treatment ultimately led to her discontinuing her diabetes medications for several months, resulting in blood glucose levels in the 400s and worsening symptoms, including frequent urination and poor sleep. After finally connecting with a new medical team, Kim experienced difficulty expressing her needs to her medical team, limiting her ability to effectively ask for a medication regimen that worked for her. Over time, she grew comfortable navigating her care with her new provider, eventually accessing insulin pens instead of needles and a continuous glucose monitor, which substantially improved her ability to track and regulate her blood sugar, reducing stress and increasing awareness of dietary impacts. However, this access was neither immediate nor seamless, underscoring how, for participants like Kim, the process of moving into rental assistance can temporarily destabilize medical continuity and impede diabetes management, even when housing conditions themselves improve. Following her initial transition into rental assistance, Kim's glucose levels decreased, with her HbA1c falling from 13.5% to 11.0%, but then rose back up to 14.0% in the year she did not receive medical care.

Counter to Kim's example, some of our participants applied their vouchers to their existing housing when they started receiving rental assistance and were able to maintain a connection with their established medical team. Lynn's story exemplifies this pathway. When her voucher was initially issued in New Haven, she faced barriers to finding a rental unit within the HUD budget and with a landlord who would accept her voucher. Upon consultation with her existing landlord and the New Haven Housing Authority, she ultimately sought to port it to the Connecticut city where she was already living, so she could remain in her same apartment and community. This process was supported by a letter from her physician, which illustrated active collaboration between Lynn and her medical team during the housing transition and underscored that her housing stability was explicitly relevant to her health care. Remaining in her current apartment meant Lynn could continue her care with her long-term provider, with whom she reported comfort discussing her health, and she maintained regular access to medications and testing supplies. Throughout her time in the research study and her transition into rental assistance, Lynn maintained well-controlled glycemic levels (HbA1c < 6.5). Her ability to remain in place preserved continuity of care and likely supported ongoing diabetes self-management during her transition into rental assistance by minimizing disruption to both her medical relationships and routines.

Transitions into rental assistance can either support or disrupt access to consistent medical care, depending on the nature of the housing change and the resources available to navigate it. Effective diabetes management relies on ongoing provider relationships, regular monitoring, and uninterrupted access to medications and supplies. Even temporary disruptions to care continuity during housing transitions may adversely affect glycemic control, whereas stability in care can reinforce sustained self-management.

#### 3.2.2. Autonomy in diabetes management

For some tenants, moving into rental assistance meant having a place of their own. Participants described this shift as increasing their sense of autonomy in managing their diabetes and daily routines. This was particularly true for participants who were doubled up or living in unstable housing situations prior to leasing up, such as Michelle and Yana.

Prior to moving into her rent-assisted apartment, Michelle lived in a women's shelter, where she reported great difficulty with diabetes management. She described how the conditions and rules in the shelter made it hard to take her insulin and test her blood sugar. As she explained, “They wanted me to take my injections in the bathroom, and I told them no, I wouldn't do that. I'll turn my back on a corner, and I'll inject myself that way. It was during the pandemic. There's 40, 50 people. It was like 13 or 14 girls in one room. That part was hard. One time, one of the staff got stuck by one of my needles. They threw my insulin away.” Once she was settled in her rent-assisted home, she noted that the ability to store her medications and test her glucose levels in a safe, clean, and private space supported her ability to manage her diabetes more consistently. She stated, “Oh, I'm not as stressed anymore. I'm more well-controlled because I can take my medicine every day without any worry.”

Yana experienced a similar shift in autonomy when she moved from living on her son's couch into her own rent-assisted apartment. After her move, she reported her health was “improving because I can focus on myself more …. Reminding myself to take my meds every day because when you're living with other people and things, a lot of things you miss because you don't want to have interactions with people … I can get up in the morning in time and just go to my medicine cabinet and take my meds and not run into someone. It's a blessing.” Beyond taking her medication regularly, Yana also reported that living in her own place allowed her to cook meals according to her own preferences, store food safely without concern that others would eat it. In her new place, she was able to prepare enough to eat over multiple days. She described the change as a “complete turnaround,” noting that she cooks more often, has improved her diet, lost weight, and stabilized her blood sugar. Yana also emphasized having her own space reduced stress and anxiety, giving her the mental and physical bandwidth to focus on her health. Yana's glycemic levels (HbA1c) decreased following her acquisition of rental assistance, ranging from 7.0% to 10.0% before her assistance and decreasing to 5.5% after her transition. These changes may reflect the increased sense of autonomy Yana described experiencing after moving into rent-assisted housing, particularly in relation to medication routines, food preparation, and stress management. Participants who transitioned into rental assistance, particularly those previously living in shelters or doubled-up arrangements where privacy, storage, and control over daily routines were limited, often described greater autonomy in their daily diabetes management routines. Having a stable home of one's own could reduce stress, facilitate safe medication storage, and support consistent glucose monitoring—conditions that strengthen adherence and create the structure necessary for effective long-term diabetes self-management.

#### 3.2.3. Quality of housing

Another way rental assistance may shape glycemic control is through its effects on housing quality. Participants in our study suggest wide variation in the impact of rental assistance on housing quality.

Some participants described their rent-assisted housing as a significant improvement over prior living arrangements, reporting that access to functioning kitchens supported their ability to manage their diabetes. However, other participants noted that the conditions in their rent-assisted units negatively affected their diabetes management. Participants cited a lack of a stove or refrigeration, or problems with pests in their new rent-assisted units, factors that caused stress and directly interfered with their diabetes management.

Lindsey was one participant who described an especially rough time with pests in her new voucher-subsidized apartment. Lindsey and her elementary-aged son moved into their rent-assisted unit after staying with family, and soon experienced difficulties with mice and cockroaches in their unit. She stated, “I was scared to cook … This is more so with the mice. The roaches, I will just kill—the mice are different. I wouldn't want to cook because I have seen them crawl on my stove or go in my stove or come out of my stove, and it didn't sit right with me.” The Housing Authority withheld rent from the landlord for a few months until the landlord provided receipts showing that the complex had been treated for pests. However, the treatment provided only temporary relief from the mice and had no effect on the roaches. Lindsey reported that this impacted how she stored her food and the meal choices she made for herself and her son, ultimately leading her to turn to microwaveable and ready-made meals instead of prioritizing the healthier cooking options she was used to. Prior to receiving rental assistance, Lindsey's diabetes was considered under fair control (HbA1c = 8.5% at baseline), but during her time in rental assistance, her HbA1c rose to uncontrolled levels (HbA1c at follow-ups ranged from 10.5 to 12.0%). It is possible that her challenges with pests played a role in this increase.

Inadequate kitchen facilities were another concern for tenants with rental assistance. When Michelle moved into her project-based family living development, she was surprised by the absence of basic kitchen appliances. Despite inspections conducted before and after move-in, the unit did not include a refrigerator or stove, and both the landlord and rental assistance agency acknowledged the issue without offering a resolution. Michelle made frequent trips outside the home to obtain meals, and during colder months, she stored food and insulin outdoors to keep them cold. She characterized this period as a daily struggle that disrupted her ability to prepare meals and maintain regular access to food. Living on a fixed income, Michelle found it financially difficult to save for these appliances, but ultimately was able to purchase a refrigerator herself. At the follow-up interview six months later, she reported that new construction at the property would provide refrigerators and stoves in the coming weeks, a change she went on to describe as transformative. During this time of transition, Michelle's HbA1c went from 7.0% to 13.5%, then lowered again to 6.5% after she had a working kitchen.

Rental assistance may influence glycemic control through its effects on housing quality, though participants’ experiences varied widely. While some moved into units that supported safe food storage and meal preparation, others encountered substandard conditions—such as pests or missing appliances—that were described as increasing stress and disrupting daily diabetes management. These findings suggest that the health impact of rental assistance depends not only on affordability and stability, but also on the material quality of the housing itself.

## 4. Discussion

In this mixed-methods longitudinal study of adults with diabetes, changes in HbA1c over two-and-a-half years, on average, did not differ significantly between participants who obtained rental assistance and those who remained unassisted. Our qualitative findings suggest that these null quantitative findings may reflect heterogeneous, context-dependent ways in which rental assistance shapes diabetes management, illuminating mechanisms through which it may support or undermine glycemic control. Some participants described rental assistance as reinforcing continuity of medical care, improving capacity for diabetes self-management, and enhancing housing quality and stability. Others reported their transition to rental assistance involved disruptions in health care access, poor housing and kitchen quality, and disruptive moves, introducing new challenges to managing their diabetes. These divergent experiences suggest that rental assistance does not function as a uniform health intervention for diabetes, but instead shapes health through contingent pathways related to continuity of medical care, autonomy of diabetes management, and housing stability and quality.

Our quantitative findings align with existing research suggesting that rental assistance may have differential implications for health across housing contexts and program types (Denary et al., 2023; Fenelon et al., 2017, 2018, 2022). Although we were unable to examine differences by rental assistance type, our overall null findings are consistent with prior national evidence suggesting that average associations across rental assistance programs may conceal important variation in diabetes outcomes across housing models (Fenelon et al., 2022). This research found improved diabetes outcomes among recipients of project-based housing relative to a waitlist control group, but did not detect these positive associations for voucher holders. A growing body of literature suggests that project-based housing may offer greater residential stability and fewer disruptions than vouchers, which often require households to relocate and navigate constrained private rental markets characterized by landlord resistance and limited unit availability (Gold, 2018). Because our sample included a mix of rental assistance types—including Housing Choice Vouchers, public and project-based housing, and other subsidized arrangements—we may be capturing an averaged effect that obscures meaningful variation by program type. More broadly, these patterns likely reflect multiple, intersecting sources of heterogeneity in how rental assistance is experienced and implemented across contexts.

Our qualitative findings reveal substantial variation in the mechanisms linking rental assistance to health, which may hinder our ability to detect an average effect of rental assistance on health outcomes. For some participants, rental assistance reduced housing-related stress and enabled stable routines supporting medication adherence, glucose monitoring, and meal preparation—pathways that prior research has linked to improved diabetes self-management (Berkowitz et al., 2018; Keene, Guo, & Murillo, 2018; Mosley-Johnson, Walker, Thakkar, et al., 2022; Vijayaraghavan et al., 2011). For others, however, transitions into rental assistance introduced new challenges that complicated diabetes management. Residential relocation frequently disrupted established medical relationships, delayed access to care, or interrupted medication regimens. Disruptions in continuity of care are known to have particularly negative effects for individuals managing diabetes, contributing to treatment delays, medication nonadherence, and increased acute care utilization, with associations with increased HbA1c levels, hospitalizations, emergency department attendance, and mortality (Chan et al., 2021; Gulliford et al., 2006). Importantly, this is a particularly vulnerable, high-risk population for whom other interventions may be needed.

These findings align with broader literature demonstrating that housing transitions—even when ultimately beneficial—can temporarily destabilize chronic disease management. In the Moving to Opportunity Study, voucher recipients with chronic health vulnerabilities did not experience the same gains from residential mobility as families without such conditions (Osypuk et al., 2024). Housing instability and forced mobility can undermine routines, heighten stress, and erode healthcare continuity, particularly among low-income and racially marginalized populations already facing structural barriers to care (Lim et al., 2017; Smith et al., 2024; Swope et al., 2022; Swope & Hernández, 2019; Whittaker et al., 2025). Because lease-up often involves relocation, recipients may gain access to new resources but lose established providers and social supports. Participants in our study who received rental assistance demonstrated a non-statistically significant trend toward improved glycemic control at the final follow-up, suggesting that potential health benefits may emerge gradually over time. The pathways linking housing stability to glycemic control—reestablishing continuity of care, rebuilding medication routines, securing reliable food access—often unfold over extended periods and may initially be offset by the disruptions associated with housing transitions before measurable metabolic improvements emerge. Our qualitative findings further suggest that the transition into rental assistance can involve a temporary period of instability, particularly when participants moved neighborhoods, changed healthcare providers, or navigated substandard housing conditions before achieving greater residential stability. Evidence on care continuity underscores that disrupted provider relationships can delay treatment, reduce adherence, and worsen glycemic control, whereas remaining in place or relocating to areas with better healthcare access may support engagement in care (Chan et al., 2021; Gulliford et al., 2006). While rental assistance may reduce long-term housing insecurity, the process of lease-up itself may contribute to a period of heightened vulnerability for individuals with diabetes.

Housing quality emerged as another critical pathway shaping glycemic outcomes. Although rental assistance programs require units to meet minimum Housing Quality Standards, participants’ experiences revealed substantial variation in access to functional kitchens, food storage, and pest-free environments—features central to diabetes self-management (Keene, Henry, et al., 2018; Mosley-Johnson, Walker, Nagavally, et al., 2022, 2022; Newman & Holupka, 2018). For participants transitioning from homelessness, shelters, or doubled-up housing, rent-assisted units often represented a marked improvement, enabling safe medication storage, regular meals, and increased autonomy over daily routines, consistent with prior qualitative studies of housing and diabetes management (Keene, Henry, et al., 2018). However, other participants described substandard conditions in rent-assisted units, including lack of appliances or persistent pest infestations, which constrained food preparation and increased reliance on ready-made or ultra-processed foods—patterns associated with poorer glycemic control (Mosley-Johnson, Walker, Nagavally, et al., 2022; Shaheen et al., 2021; Vijayaraghavan et al., 2011).

Participants receiving Housing Choice Vouchers appeared particularly vulnerable to these challenges. Consistent with existing research on voucher use, some participants described pressure to accept units that met program requirements but did not meet their health-related needs, reflecting constrained choice in tight rental markets (Ellen, 2020; Ellen et al., 2023; Engel et al., 2025; Rosen, 2020). Voucher holders may prioritize securing any unit within the allowable time frame to avoid losing assistance, even when housing quality is suboptimal (Buron et al., 2007; Engel et al., 2025). In this context, rental assistance may alleviate financial strain while simultaneously exposing individuals to housing or neighborhood conditions that undermine their diabetes management.

Together, these findings underscore the importance of complementary supports during transitions into rental assistance. Residential moves are inherently disruptive, and for individuals with diabetes, even short-term disruptions to care continuity or daily routines can have clinically meaningful consequences (Berkowitz et al., 2013, 2018, 2025). Case management or navigation services that explicitly integrate housing transitions with medical care—such as assistance transferring providers, securing medication refills, or coordinating follow-up appointments—may help mitigate these risks. Prior research suggests that health-informed housing supports can improve access to care and reduce unmet medical need, indicating a promising avenue for enhancing the health impacts of rental assistance (Fenelon & Chen, 2025; Gonzalez-Lopez et al., 2024; Sanjeevi et al., 2025).

### 4.1. Limitations

Several limitations should be considered when interpreting these findings. First, the analytic sample was smaller than originally planned, which limited statistical power to detect modest effects of rental assistance on HbA1c. We initially aimed to enroll 300 participants, which—assuming 75% retention and 70% uptake of rental assistance—would have provided sufficient transitions to detect a 0.5% change in HbA1c with 90% power, adequately powering our main and subgroup analyses. However, despite high retention rates, the sample was limited by modest recruitment (n = 113) and multiple deaths during follow-up, constraining study power and possibly limiting our ability to identify clinically meaningful effects. Second, our sample size prevented us from disaggregating effects by rental assistance type, despite evidence that different program types (e.g., project-based vs voucher-based) may have distinct implications for housing stability and health (Fenelon et al., 2022). Third, most participants (71%) had fairly well-controlled diabetes at baseline (HbA1c < 9), indicating less room for improvement following rental transitions. Finally, participants moved in and out of rental assistance during the study period, reflecting real-world housing instability but complicating causal attribution.

The timing of the study also warrants careful consideration. Data collection began in November 2020, during the COVID-19 pandemic—a period marked by widespread disruptions to healthcare delivery, heightened stress, and the introduction of multiple emergency support programs, including expanded food assistance and emergency rental aid. Concurrent changes in diabetes treatment, including the increasing availability of GLP-1 receptor agonists, may have influenced HbA1c trajectories independently of housing conditions. These overlapping structural and clinical shifts complicate efforts to isolate the effects of rental assistance alone.

Finally, improvements in glycemic control may lag behind receipt of rental assistance. Because participants entered rental assistance at different points during the study period, we were unable to systematically assess how time since lease-up shaped HbA1c trajectories. However, these findings suggest that the health impacts of rental assistance may be temporally dynamic, with short-term disruptions potentially obscuring longer-term benefits. Longer-term follow-up may be necessary to fully capture the downstream metabolic effects of improved housing affordability and stability.

## 5. Conclusion

Our findings suggest that rental assistance does not operate as a one-size-fits-all intervention for diabetes, but rather has varied implications for individuals and contexts. Although we did not identify a statistically significant impact of rental assistance on glycemic control, our study may not have been powered to detect such an effect. Further, our qualitative findings point to important variation in how rental assistance shapes health. For many individuals, access to stable and affordable housing clearly shaped their ability to navigate the daily demands of living with diabetes, reducing housing-related stressors and creating conditions more conducive to consistent self-management. For others, the transition to rental assistance sometimes introduced disruptions—such as residential moves that disrupted continuity with established medical providers—temporarily complicating diabetes management. In addition, deficiencies in some rent-assisted units, particularly related to kitchens, appliances, or pest control, created practical and emotional barriers to preparing food, storing insulin, and maintaining daily diabetes routines.

These results suggest the health implications of rental assistance may depend on how assistance is delivered, the quality and location of housing obtained, and the extent to which transitions into assisted housing are supported. Policies that seek to leverage housing as a platform for chronic disease management may need to pair rental assistance with greater attention to housing quality, residential stability, and integration with healthcare and social services in order to more fully realize its potential to support diabetes management and improve health over time.

## Supplementary Material

Supplemtary Data: Figure 1

## Figures and Tables

**Table 1:** Participant characteristics at baseline.

Baseline Characteristic	Total Sample (n = 113)	Did not receive Rental Assistance (n = 71)	Received Rental Assistance (n = 42)	*p*-value

**Age at Baseline, mean (SD)**	51.3 (11.3)	51.6 (9.7)	50.8 (13.7)	0.69
**Race, n (%)**				0.64
African American	65 (57.5%)	39 (54.9%)	26 (61.9%)	
White	30 (26.5%)	21 (29.6%)	9 (21.4%)	
Other	18 (15.9%)	11 (15.5%)	7 (16.7%)	
**Ethnicity, n (%)**				0.99
Non-Hispanic	85 (75.2%)	53 (74.6%)	32 (76.2%)	
Hispanic	28 (24.8%)	18 (25.4%)	10 (23.8%)	
**Gender, n (%)**				0.10
Man	42 (37.2%)	31 (43.7%)	11 (26.2%)	
Woman	71 (62.8%)	40 (56.3%)	31 (73.8%)	
**Employment in the Last Six Months, n (%)**				0.22
No	74 (65.5%)	43 (60.6%)	31 (73.8%)	
Yes	39 (34.5%)	28 (39.4%)	11 (26.2%)	
**Child Living at Home, n (%)**				0.67
No	95 (84.1%)	61 (85.9%)	34 (81.0%)	
Yes	18 (15.9%)	10 (14.1%)	8 (19.0%)	
**Previously Incarcerated, n (%)**				0.70
No	66 (58.4%)	40 (56.3%)	26 (61.9%)	
Yes	47 (41.6%)	31 (43.7%)	26 (38.1%)	
**Years since Diabetes Diagnosis (baseline)**				0.32
0–4 years ago	43 (38.1%)	27 (38.0%)	16 (38.1%)	
5–10 years ago	27 (23.9%)	14 (19.7%)	13 (31.0%)	
10+ years ago	43 (38.1%)	30 (42.3%)	13 (31.0%)	
**HbA1c at Baseline, mean (SD)**	8.0 (2.2)	8.2 (2.3)	7.7 (2.0)	0.23
**Controlled Diabetes at Baseline, n (%)**				0.30
Controlled (HbA1c < 9)	81 (71.7%)	48 (67.6%)	33 (78.6%)	
Uncontrolled (HbA1c ≥ 9)	32 (28.3%)	23 (32.4%)	9 (21.4%)	

**Note:** P-values were calculated to compare baseline characteristics of participants who did not receive rental assistance to those who received rental assistance at any point during the study using two-sample t-tests (continuous variables) and chi-square tests (categorical variables). (HbA1c = hemoglobin A1c.)

**Table 2:** Least Mean Square Estimates of Change of HbA1c Levels from Baseline by Rental Assistance Status and Visit, and Pairwise Contrasts (Yes vs. No).

Follow Up Visit	Rental Assistance Status at Follow Up	Least Mean Square Estimates	Contrast (Yes–No)	*p*

**Visit 1**	No	−0.32 (−0.65, 0.02)	−0.15 [−0.87, 0.57]	0.689
	Yes	−0.46 (−1.16, 0.23)		
**Visit 2**	No	0.06 (−0.29, 0.42)	−0.13 [−0.77, 0.52]	0.701
	Yes	−0.06 (−0.66, 0.54)		
**Visit 3**	No	−0.07 (−0.44, 0.30)	−0.04 [−0.64, 0.56]	0.887
	Yes	−0.11 (−0.66, 0.43)		
**Visit 4**	No	0.09 (−0.28, 0.45)	−0.01[−0.6, 0.59]	0.982
	Yes	0.08 (−0.45, 0.62)		
**Visit 5**	No	−0.09 (−0.49, 0.31)	−0.50 [−1.05, 0.06]	0.079
	Yes	−0.58 (−1.05, −0.12)		

**Note:** All follow-up visits occurred in 6-month intervals. Estimates are adjusted marginal mean differences in change in HbA1c from baseline (Yes – No rental assistance), averaged over age at baseline, baseline years since diabetes diagnosis, and incarceration history.

## Appendix A. Supplementary data

Supplementary data to this article can be found online at https://doi.org/10.1016/j.ssmqr.2026.100817.
