# Supplementary figures and images for: Rental assistance impacts on diabetes: Insights from a longitudinal mixed-methods analysis
Source: SSM Qual Res Health. Author manuscript; Available in PMC 2026 Jul 31. (PMC13416935; doi:10.1016/j.ssmqr.2026.100817)

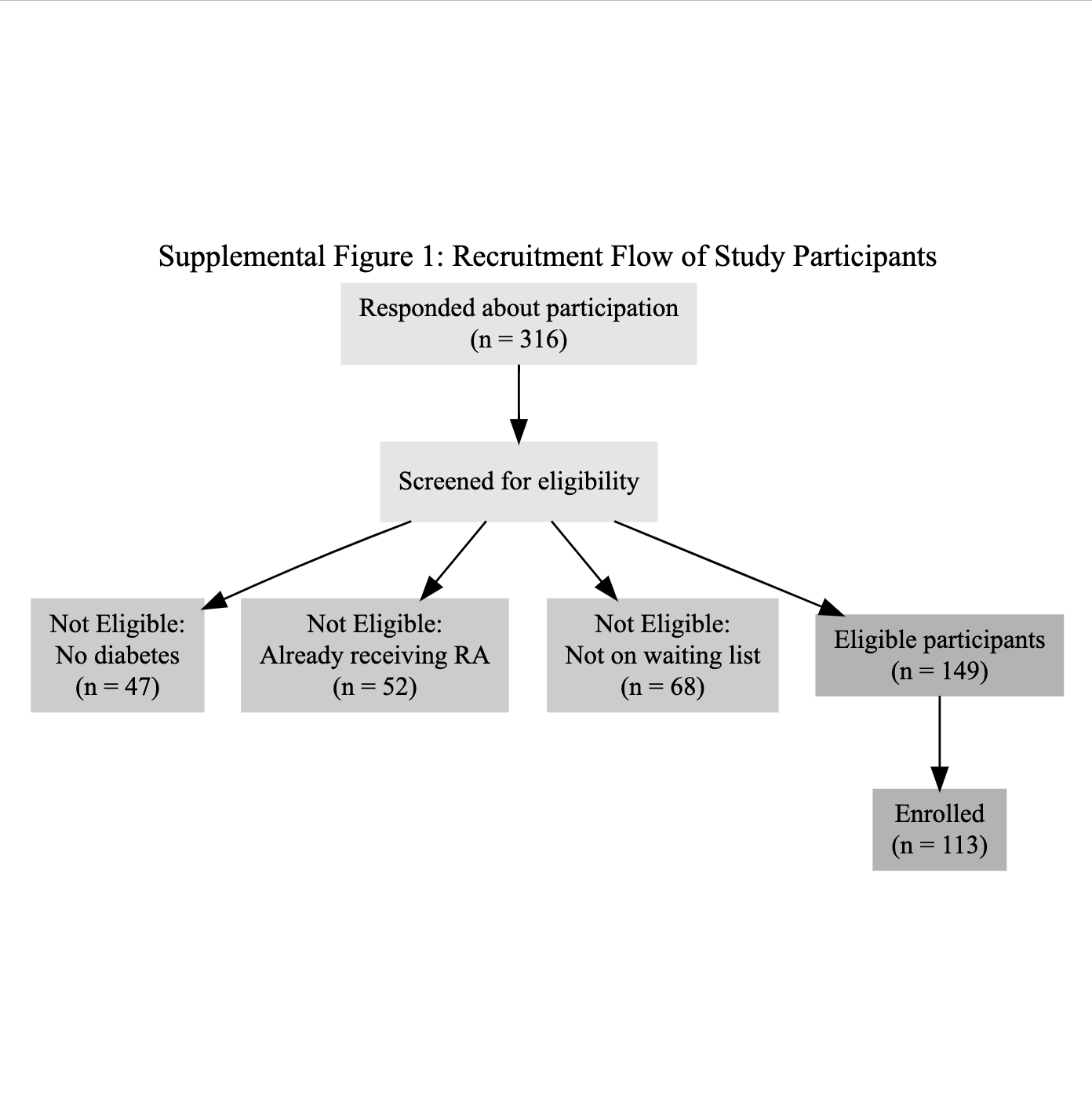

Supplement: Supplemtary Data: Figure 1 [file NIHMS2194397-supplement-Supplemtary_Data__Figure_1.docx]
